# The American Medical Association

**Published:** 1883-07

**Authors:** 


### THE AMERICAN MEDICAL ASSOCIATION.

There is nothing about which men quarrel so persistently and bitterly as over questions of morals and ethics. The truth of this is abundantly demonstrated by the present controversy between the two factions into which the medical profession in this State is now unfortunately divided. In the May number of the INDEPENDENT PRACTITIONER we expressed the opinion that the only way in which the differences of opinion which had even then degenerated into an altercation could be satisfactorily settled, was by an appeal to the American Medical Association, provided there could be a full and fair expression of professional preferences. But that august body has not chosen to hear the other side at all. At its late meeting it took the unheard-of step of requiring all permanent and delegate members to sign a declaration binding them to maintain the old code of ethics, and refusing a seat to all who would not thus pledge themseves in advance.

It is quite legitimate to make new members promise a compliance with the rules and regulations of the body to which they seek admission, but to endeavor to force old members to sign such a declaration under durance, savors rather too much of the fagot and the block. There can be no advance without free discussion, and when this is denied a respectable minority, they are certain to obtain the sympathy of all who hate oppression and tyranny. Reasonable men are not convinced by clubs, and persecution has always strengthened the side of the oppressed, because all candid men love fair play. No one whose mind is open to conviction will consciously promise that his opinion will not change, or that he will not to-morrow take a step which he may then be convinced is an advance. If its code of ethics or any particular method of practice is too sacred for open discussion, medicine will soon find itself far in the rear of human progress. We are not now, nor have we heretofore been, an advocate of the proposed changes, but candor compels the admission that a few such instances of intolerance of question and enquiry will go far toward creating a prejudice against a body that should possess the confidence of every medical man.

